# Antibiotic use amongst pregnant women in a public hospital in KwaZulu-Natal

**DOI:** 10.4102/hsag.v26i0.1516

**Published:** 2021-05-31

**Authors:** Sasha Naidoo, Varsha Bangalee, Frasia Oosthuizen

**Affiliations:** 1 Discipline of Pharmaceutical Sciences, College of Health Sciences, Faculty of Pharmacy, University of KwaZulu-Natal, Durban, South Africa

**Keywords:** pregnancy, antibiotics, pharmacovigilance, teratogenicity, public healthcare

## Abstract

**Background:**

Antibiotics are amongst the more frequently prescribed medicines in pregnant women and the use of antibiotics is increasing. However, with limited studies available in this population, the safe use of antibiotics in pregnancy remains a concern.

**Aim:**

To evaluate the use of antibiotics amongst pregnant women attending a public health care facility. The main objective of this study was to quantify the types of antibiotics used in pregnant women.

**Setting:**

A public hospital classified as a referral hospital located in Durban, KwaZulu-Natal.

**Methods:**

Demographic and treatment information of women were collected retrospectively from January 2019 to July 2019. A total of 184 pregnant patients, who received antibiotic therapy, were included in this study. Descriptive and analytical measures were used to analyse both patient demographics and treatment variables.

**Results:**

A total of 416 antibiotic prescriptions, issued to 184 patients, were reviewed. Penicillins (39.7%), macrolides (13.0%) and combination penicillin- and beta-lactam inhibitors (12.3%) were reported as the most commonly prescribed antibiotics. Rifamycin (2.9%), hydrazides (2.2%) and aminoglycosides (1.9%) were less frequently prescribed. Most antibiotics were prescribed for diseases of the circulatory system (36.1%).

**Conclusion:**

Several classes of antibiotics were used in pregnancy despite the lack of available safety data and clinical evidence. Informing women of the potential side effects and keeping abreast with new information played an important role in the safe, rational and effective use of medicines that contributed to improving maternal health.

## Introduction

Antibiotic use in pregnancy is increasing (Broe et al. 2014) and antibiotics account for a significant percentage of all medication that is prescribed in pregnancy (Kuperman & Koren 2016). According to several studies, one out of every four pregnant women is prescribed an antibiotic (Bookstaver et al. 2015; Santos, Oraichi & Bérard 2010; Stokholm et al. 2013).

The use of antibiotics in pregnancy is attributed to the fact that pregnancy predisposes the body to infection. Hormone fluctuations and changes in immunity are the primary causes for increasing the body’s susceptibility to infection (Cherney 2016). Genitourinary infections are the most frequently occurring illness contributing to the upsurge in antibiotic use (Lee et al. 2019, 2020). According to a German study, 72% of antibiotic treatment is a result of vaginal candidiasis and urinary and respiratory tract infections (Amann et al. 2006).

Antibiotic safety is an essential factor when considering its use in pregnancy. Antibiotics may be teratogenic and may have both short- or long-term effects on the foetus (Vidal et al. 2013). Several modifying influences may affect the severity of teratogenicity. These include the gestational period of the pregnancy (ed. Rossiter 2020), the dose and duration of therapy, genetic predisposition, environmental factors and the degree of drug transfer across the placenta (Amann et al. 2006; Ledger & Blaser 2013; Nahum, Uhl & Kennedy 2006).

There is inadequate evidence regarding the safety of antibiotics used in pregnancy because of ethical limitations (Bookstaver et al. 2015). Clinical trials amongst the pregnant population are restricted and hence, the teratogenicity of various medicines is unknown (Crider et al. 2009). A review regarding antibiotic use in pregnancy found that only approximately 10% of medicines used in pregnancy are supported by safety data (Adam, Polifka & Friedman 2011). In this study, antibiotics were classified according to the Food and Drug Administration (FDA) ‘A to X’ safety categories. Category A are the safest, whilst medicines in category X should be completely avoided (Food and Drug Administration 2017). Category B are drugs where animal studies may or may not have revealed evidence of harm to the foetus; however, there are no adequate and well-controlled studies in pregnant women. Category C are drugs where animal studies may or may not have been conducted to show adverse effects, but there are no adequate and well-controlled studies in pregnant women. Category D are drugs where adequate, well-controlled studies in animals or pregnant women have demonstrated positive evidence of foetal abnormalities. However, the benefits of therapy may outweigh the potential risk (Food and Drug Administration 2019).

Currently, there is no standardised approach to the rational, safe and effective use of antibiotics in pregnant women. Ethical concerns limit the availability of clinical evidence that is available in this population. Prescribers use a risk-benefit approach to treat pregnant patients based on the trimester of pregnancy, the severity of the illness and potential foetal risk. The emerging crisis of antibiotic resistance significantly affects the use of antibiotics in pregnant women with the overuse and misuse of these drugs being key drivers of antimicrobial resistance (AMR). The epidemic of AMR is changing the way antibiotics are used, increasing mortality and morbidity and greatly increasing the cost of healthcare. The management and use of antibiotics have clinical, economic and environmental implications.

The primary aim of this study was to evaluate the use of antibiotics amongst pregnant women attending a public healthcare facility. The main objective of this study was to quantify the types of antibiotics used in pregnant women. The secondary objectives were to comment on the trends, rationale and safety profile of the prescribed antibiotics.

## Research methods and design

### Study design and setting

This retrospective study was conducted over 7 months (from January 2019 to July 2019) at a tertiary and quaternary public hospital, centrally located in Durban, KwaZulu-Natal (Department of Health 2017).

### Study population and sampling strategy

The selected population consisted of both inpatients and outpatients. Participants included in this study met the following eligibility criteria:

- 18 years of age or above
- female and pregnant
- treated with an antibiotic as an inpatient or outpatient.

Details around antibiotic use, diagnosis, patient demographic information and human immunodeficiency virus (HIV) status were recorded. A probability sampling method was adopted, which included every potential participant who visited the hospital from January 2019 to July 2019 and met the eligibility criteria, yielding a sample size of 184 participants. This sample size was large enough to allow for a detailed analysis of antibiotic usage as each patient received an average of two to three prescriptions, yielding a total of 416 prescriptions.

### Data collection

Data were obtained from a software programme called MediTech®. MediTech® electronically stores patient files and treatment information. A pre-designed data collection tool was used to record available demographic and treatment information from patient files (Appendix 1). The template was developed according to guidelines by the World Health Organisation (WHO) that included key antimicrobial use indicators designed specifically for the collection of data in hospitals (USAID 2012). Data on the following elements were recorded:

- demographics (age, ethnicity, risk of pregnancy and HIV status)
- antibiotic name
- dose
- route of administration
- frequency of administration
- duration of treatment.

All information collected was kept confidential. Patient names were coded and records were kept in a password protected device. Individual patients’ written informed consent for the data collection was not needed, as only anonymised, retrospective data were extracted.

### Data analysis

Descriptive and analytical measures were used to describe both patient demographics and treatment variables. Measures of central tendency such as the frequency, mean and median were calculated. Antibiotic usage patterns amongst the participants were then identified. The quantitative data values are represented in frequency distribution tables by grouping them into categories and in a frequency distribution bar chart.

The linear relationship between the patient demographics and treatment variables was established using correlation analysis. The relationship between treatment duration, age, HIV co-infection and the number of prescriptions was investigated using the Chi-square test. The level of significance was set at α = 0.05.

### Eliminating bias

Firstly, the probability for biased selection was eliminated as a probability sampling method was adopted, which included every pregnant woman who visited the hospital from January 2019 to July 2019 and who met the criteria. Secondly, data were collected using a pre-designed tool, and the extraction of data was not performed by medical staff who prescribed or dispensed the antibiotics.

### Ethical considerations

Ethical approval was sought from the relevant bodies; the Biomedical Research and Ethics Committee (BREC) at the University of KwaZulu-Natal (UKZN) (BE330/19), KZN Department of Health (KZN-DoH) and the Head of Department and Chief Executive Officer (CEO) at the study site.

### Results

#### Demographic and general characteristics

Patient demographic data for 184 pregnant women are shown in Table 1. Less than 10% of the study population were between the age of 18 and 20. The majority of women were evenly distributed between the other two remaining age groups (20–29, 30–42). Almost 92% of the study population were black African women, with a small percentage being Asian women (4.9%), white women (1.6%) and women of mixed race (1.1%). Many of the women (82.1%) were reportedly single, with 12% being married, whilst the marital status of the remaining women (6.0%) was not reported. A significant percentage (63.1%) of women were in their third trimester of pregnancy, with less than 35% of pregnancies being categorised as high risk (women with co-morbidities). A large percentage of women (45.7%) who were included in the study were co-infected with HIV. Most women visited this tertiary facility between two and five times during their pregnancy.

**TABLE 1:** Characteristic and demographic data of participants, *n* = 184.

Demographics	Frequency (*N*)	%
**Age**		
18–20	14	7.6
20–29	86	46.7
30–42	84	45.7
**Race**		
Black people	169	91.8
Asian people	9	4.9
White people	3	1.6
Mixed race people	2	1.1
Others	1	0.5
**Marital status**		
Married	22	12.0
Single	151	82.1
Not reported	11	6.0
**Gestational weeks**		
0–13	0	0.0
14–26	3	1.6
27–40	116	63.0
Not reported	65	35.3
**High-risk pregnancy**		
Yes	63	34.2
No	121	65.8
**HIV**		
**Co-infection**		
Yes	84	45.7
No	100	54.3
**Inpatient days**		
< 3	46	25.0
4–14	88	47.8
18–88	50	27.2
**Outpatient visits**		
≤ 1	57	31.0
2–5	84	45.7
5–15	43	23.4

HIV, human immunodeficiency virus.

#### Correlations investigated

Demographic variables correlated with the duration of antibiotic treatment included the following:

- age group (> 20, *p* = 0.0009, 20–29, *p* = 0.017, 30–42, *p* = 0.03)
- gestational week (*p* = 0.4)
- HIV co-infection (*p* = 0.9)
- high-risk pregnancy (*p* = 0.9).

#### Quantifying antibiotic usage in pregnancy

A description of the different antibiotic classes and their prescribed frequencies are shown in Table 2. A total of 416 prescriptions were dispensed to 184 patients, with each woman receiving more than one course of antibiotic therapy during their pregnancy. Most women received an average of one to two courses, with 8.7% of patients receiving more than four prescriptions during their pregnancy.

**TABLE 2:** Frequencies of the prescribed Food and Drug Administration categories and antibiotic classes, *n* = 184.

FDA categories	Antibiotic class	Frequency	%
**B**	Combination of penicillin and beta-lactam inhibitors	51	12.3
	Carbapenems	26	6.3
	(except imipenem-category C)		
	Glycopeptides	3	0.7
	Imidazoles	20	4.8
	Lincosamides	3	0.7
	Macrolides	54	13.0
	Nitrofurantoin	34	8.1
	Penicillins	165	39.7
	Third generation cephalosporins	13	3.1
	Topical antibiotics	1	0.2
Total	-	370	88.9
**C**	Amphenicols	3	0.7
	Fluoroquinolones	5	1.2
	Hydrazides	9	2.2
	Polymyxins	2	0.5
	Rifamycins	12	2.9
	Trimethoprim – sulphonamide	7	1.7
	combinations	**-**	**-**
Total	-	38	9.2
**D**	Aminoglycosides	**-**	**-**
	(except gentamycin – category C)	8	1.9
Total	-	8	1.9
**Total**	**-**	**416**	**100.0**

Whilst the Pregnancy and Lactation Labelling Rule (PLLR) has come into effect for all new drugs registered in the United States of America and is being phased in for older drugs, medication use in this study was classed according to the ‘A to X’ categories of the FDA (Feibus 2008; Food and Drug Administration 2017, 2019). A large percentage (88.9%) of prescriptions were Food and Drug Administration (FDA) category B antibiotics with the remaining 11.1% being categories C and D.

Penicillins were the most frequent class (39.7%) of antibiotics prescribed to pregnant women followed by the macrolides (category B) (13.0%) and combination penicillin- and beta-lactam inhibitors (category B) (12.3%). Nitrofurantoin (8.1%) and carbapenems (6.3%) were also amongst the commonly used antibiotics, although they were slightly less prescribed in comparison to macrolides and beta-lactams. Other antibiotics such as rifamycin, hydrazides, aminoglycosides, fluoroquinolones and trimethoprim-sulphonamide combinations were sparingly prescribed.

#### Category B antibiotics commonly prescribed

The five most frequently prescribed category B antibiotic classes (drugs where animal studies may or may not have revealed evidence of harm to the foetus) and their respective dosing characteristics, including the route of administration and indication, are described in Table 3. Phenoxymethylpenicillin (pen V) was the most prescribed penicillin antibiotic (74.5%). Among the combination penicillins, amoxicillin-clavulanic acid was commonly used (21.8%). Azithromycin was the only prescribed macrolide and meropenem was one of the frequently prescribed carbapenems.

**TABLE 3:** Characteristics of frequently prescribed Food and Drug Administration category B antibiotic classes.

Antibiotic class	Description	Strength (mg)	Dosing frequency	Average duration (Days)	Route of administration	Most common indication by disease category	No of prescriptions
Penicillins	Amoxicillin	500, 1000	8 hourly	4	Oral IM†	Diseases of the circulatory system	24
	Flucloxacillin	250–500	6 hourly	6	Oral		8
	Pen V‡	125, 250	12 hourly	9	Oral		123
	Ampicillin	1000	8 hourly	4	IV§		1
	Benzathine benzylpenicillin	2400	Once daily	1	IM		9
Total	-	-	-	-	-	-	165
Macrolides	Azithromycin	250, 500 and 1000	Once daily	3	IV	Diseases of the circulatory system	54
					Oral		
Total	-	-	-	-	-	-	54
Combination of penicillins and beta-lactam inhibitors	Amoxicillin/clavulanic acid	375	8 hourly	4	Oral	Diseases of the circulatory system	36
		1000	12 hourly		IV		
		1200	8 hourly		-		
	Piperacillin/tazobactam	2250	8 hourly	3	IV		15
		4500					
Total	-	-	-	-	-	-	51
Nitrofurantoin	Nitrofurantoin	50, 100	6 hourly	5	Oral	Diseases of the genitourinary system	34
Total	-	-	-	-	-	-	34
Beta-lactam carbapenems	Imipenem/ cilastatin	500 and 1000	6–8 hourly	4	IV	Diseases of the genitourinary system	5
	Meropenem	500 and 1000	6–8 hourly	3			21
Total	-	-	-	-	-	-	26

IV, Intravenous; IM, Intramuscular.

† , Intramuscular.

‡ , Pen V – Phenoxymethylpenicillin.

§ , Intravenous.

The strength, dosing frequency and average duration varied amongst the different classes with most women receiving oral or intravenous (IV) doses of administration, whilst a few received treatment intramuscularly (IM).

Most antibiotics were given for 7 days or less. Phenoxymethylpenicillin was an exception as the average duration for this antibiotic was 9 days. The remaining penicillin classes, nitrofurantoin and carbapenems were commonly used for 4–7 days. Macrolides were frequently prescribed for less than or equal to 3 days.

#### Category C and D antibiotics commonly prescribed

Table 4 shows the prescribed category C and D antibiotics (drugs where animal studies may or may not have been conducted to show adverse effects, but there are no adequate and well-controlled studies in pregnant women) with their subsequent characteristics. Rifamycins (2.9%), hydrazides (2.2%) and aminoglycosides (1.9%) were the three most commonly prescribed category C and D drug classes. Amikacin was used more often in comparison to gentamicin. Quinolones were prescribed to five patients. Chloramphenicol, as a topical antibiotic, was prescribed to three patients. Colistin was used in two patients.

**TABLE 4:** Characteristics of frequently prescribed Food and Drug Administration category C and D antibiotic classes.

Antibiotic class	Description	Strength and dosing frequency (mg)	Dosing frequency	Average duration (Days)	Route of administration	Most common indication by disease category	No. of prescriptions
Rifamycins	Rifampicin	450–600	Daily	7	Oral	Diseases of the respiratory system	12
Total	-	-	-	-	-	-	12
Hydrazides	Isoniazid	100–300	Daily	23	Oral	Diseases of the respiratory system	9
Total	-	-	-	-	-	-	9
Aminoglycosides	Amikacin	250, 500, 900 and 1000	Once daily	3	IV	Diseases of the genitourinary system	7
	Gentamicin	240		3			1
Total	-	-	-	-	-	--	8
Trimethoprim – sulphonamide combinations	Co-trimoxazole	160/800	12 hourly	22	Oral	Diseases of the genitourinary system	7
		320/1600	6 hourly				
Total	-	-	-	-	-	-	7
Fluoroquinolones	Ciprofloxacin	500	12 hourly	5	Oral	Diseases of the circulatory system	5
					IV		
Total	-	-	-	-	-	-	5
Amphenicols	Chloramphenicol	-	12 hourly	28	Topical	Diseases of the skin and tissue	3
Total	-	-	-	-	-	-	3
Polymyxins	Colistimethate (Colistin)	80	12 hourly	8	IV	Other specified diseases complicating pregnancy and child birth that were not mentioned	2
		160	8 hourly				
Total	-	-	-	-	-	-	2

IV, Intravenous.

More than 67% of the antimycobacterial agents (rifamycins and hydrazides) were dispensed for periods longer than 8 days. Half of the women who were treated with aminoglycosides received the drug for 3 days or less, whilst the other half received it for 4–7 days. Fluoroquinolones were mostly prescribed for a short period of fewer than 5 days. Trimethoprim-sulphonamide combinations were dispensed to seven patients, with most patients receiving treatment for approximately 20 days.

#### Disease categories

**FIGURE 1:**
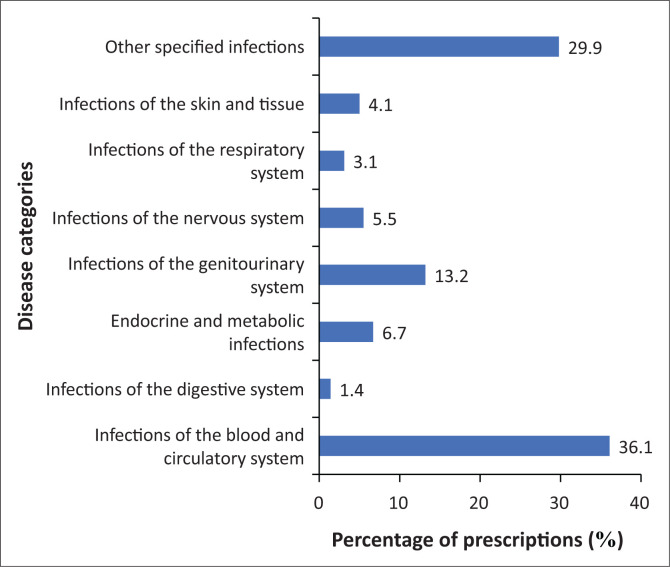
The percentage of prescriptions allocated to each disease category.

Figure 1 shows the percentage of prescriptions that were dispensed for the main categories of infections. The majority of prescriptions were for infections of the blood and circulatory system (36.1%), followed by other specified infections (29.9%) and genitourinary infections (13.2%). Endocrine and metabolic infections (6.7%) and infections of the nervous system (5.5%) contributed to a smaller number of prescriptions.

## Discussion

The primary aim of this study was to evaluate the use of antibiotics amongst pregnant women attending a public healthcare facility.

Safety and efficacy information of antibiotics is usually very limited in pregnancy because of ethical implications around clinical testing in pregnant females. This study classifies the safe use of antibiotics according to the FDA safety categories.

A significant correlation was established between the age of a patient and the duration of the antibiotic course. As the age increased, the duration of therapy increased (> 20, *p* = 0.0009, 20–29, *p* = 0.017, 30–42, *p* = 0.03). No previous studies have investigated this relationship. It is possible that with increased age, the change in pregnancy hormone levels and the patients’ immune response may contribute to a longer recovery and hence the longer duration of treatment. However, this may differ amongst individuals and various studies (Erickson & Banks 2019). This explanation, therefore, cannot be generalised.

The prevalence of the HIV in South Africa remains high (Cohn & Clark 2014; Ntlantsana, Hift & Mphatswe 2019) and considering the large percentage of HIV-positive women in this study, it was expected that the duration of antibiotic therapy and the number of prescriptions dispensed to these women would be higher compared with HIV-negative pregnant women. There was, however, no significant correlation between HIV status and antibiotic use or treatment duration (*p* = 0.9). This might indicate the positive impact of antiretroviral (ARV) therapy on decreasing the possible risk of infection in HIV-positive individuals. Similarly, these results were found in KwaZulu-Natal and Nigeria, although this explanation cannot be extrapolated to the general population and is limited to this study (Calder et al. 2020; Mepham et al. 2011; Ntlantsana et al. 2019).

The gestational period is an important factor that influences antibiotic therapy. Multiple studies advise against the use of drugs in the first trimester as the foetus is still developing (Knothe & Dette 1986; Shaabaan et al. 2020). In our study, no patients were treated with an antibiotic in their first trimester of pregnancy. The majority of patients received treatment in their third trimester, with only a few women receiving treatment in their second trimester. A recent Cochrane review evaluated the use of antibiotics in the second and third trimesters (Thinkhamrop et al. 2015). The review established that there was no increased risk of congenital abnormality between the two trimesters. However, the authors concluded that there was insufficient evidence to fully evaluate possible foetal harm (Thinkhamrop et al. 2015).

As to be expected, almost two thirds of the study population received a beta-lactamase-sensitive agent with phenoxymethylpenicillin (category B), used most commonly because of its longstanding safety record. These antibiotics remain widely prescribed in pregnancy (Bookstaver et al. 2015; Heikkilä & Erkkola 1994a). Literature indicates that beta-lactams represent approximately 65% of all antibiotic treatment used during pregnancy, with penicillins accounting for 30% (Bookstaver et al. 2015; Heikkilä & Erkkola 1994a). Similar results were obtained from our study, indicating that 61% of all antibiotics prescribed to pregnant women belonged to a beta-lactam class. We, however, found a slightly higher percentage (39.7%) of women being treated with penicillin in comparison to the northern European study (Heikkila et al. 1992). The results from our study are, therefore, consistent with the current trends of antibiotic therapy in pregnancy (Anitha et al. 2018).

Our study found that the duration for penicillin treatment ranged from 1 to 9 days and was typically within the normal prescribing guidelines. However, the hydrophilic nature of these compounds makes them more water soluble and hence the potential for lower serum concentrations, because of more rapid renal clearance during pregnancy. Observational studies have documented these changes resulting in lower concentrations, especially in the third trimester (Anderson 2005; Frederiksen 2001). These changes may lead to pharmacokinetic alterations that require dose adjustments or careful monitoring and assessment (Costantine 2014). Dose adjustments in this study were not evident.

As resistance to commonly used drugs increases, the use of other antibiotic classes will need to be sought where the safety profile has not yet been established (Lamont, Blogg & Lamont 2014; Walsh & Wright 2005). In this study, the impact of bacterial resistance was already apparent in that some women could not be administered first-line agents (penicillin monotherapy) but were placed on combination penicillins, cephalosporins and carbapenems. Observantly, combination penicillins- and beta-lactam inhibitors were used in 12% of patients. In addition, two patients were administered colistin, a drug that is not registered in South Africa and reserved for highly resistant patients (Mendelson et al. 2018).

Prescribing macrolide during pregnancy is not uncommon as similar results have been reported elsewhere (Dinur et al. 2013; Fan et al. 2020; Ramsey et al. 2003; Sarkar et al. 2006). The use of macrolides (category B) in pregnancy is, however, a growing concern (Fan et al. 2020). Significantly, a recent study by Fan et al. followed 104 605 children from birth to 14 years of age and it was concluded that prescribing macrolides in any trimester was associated with an increased risk of genital malformation (Fan et al. 2020), whereas a previous cohort of 1033 women exposed to macrolides (erythromycin, azithromycin, clarithromycin or roxithromycin) reported that there was no association with this drug and the development of major abnormalities in the foetus (Dinur et al. 2013).

This study also found that nitrofurantoin (category B), an antibiotic specific for the urinary system, was also commonly prescribed. Some studies reported no severe malformation during pregnancy, whilst others found conflicting evidence (Crider et al. 2009; Van de Mheen et al. 2014). A meta-analysis consisting of eight studies did not demonstrate any link between nitrofurantoin exposure in women and major congenital malformation (Crider et al. 2009), but other studies indicated that nitrofurantoin might increase the risk of haemolytic anaemia in pregnant patients with severe glucose-6-phosphate dehydrogenase deficiency (Van de Mheen et al. 2014). The National Birth Defects Prevention Study (NBDPS) found a significant association between nitrofurantoin use during pregnancy and cleft lip and palate (Crider et al. 2009). These adverse effects have not been commonly reported and nitrofurantoin remains an option for treatment and prevention of urinary tract infections in pregnant women, although because of its potential to cause harm, its use should be reconsidered (Van de Mheen et al. 2014).

Two carbapenems, meropenem and imipenem-cilastatin (category C), were used in this study. Carbapenems are broad-spectrum beta-lactams and are typically reserved for infections that are resistant to penicillin and cephalosporin antibiotic therapy (Blanca-Lopez et al. 2019; Heikkilä & Erkkola 1994a). The use of these antibiotics during pregnancy was minimal, indicating that they were possibly the only viable option available despite the paucity of safety data (Heikkilä et al. 1992; Mendelson et al. 2018).

Approximately 11% of all antibiotics used were category C or D agents. Anti-mycobacterial agents, specifically the class of hydrazides and rifamycin, represented about 5% of the category C drugs. This is expected as just less than half of this study population are HIV-positive who are also often on treatment for tuberculosis (TB) (Loto & Awowole 2012). The burden of TB has increased and transversed with the high incidence of HIV, as 75% of TB patients are HIV-infected. Tuberculosis may be prevalent during pregnancy as a result of immunological changes, stress and poor nutrition, particularly if there are an immunodeficiency and a co-existing disease. Studies have indicated that despite women being on combination ARV therapy, signs of TB may still appear. The WHO recommendation for treatment of TB in pregnant women is the same as for non-pregnant women and is based on standard therapy for 6 months, prolonged to a minimum of 9 months (Adhikari 2009).

Restricted use amongst the other category C and D antibiotic classes was observed and anticipated as a result of limited safety data and the possible teratogenic effects it may have on the foetus. A very small percentage of women in this study received these antibiotics as aminoglycosides, fluoroquinolones, chloramphenicol and trimethoprim-sulphonamide combinations are generally contraindicated in pregnancy unless the benefit of the drug exceeds the risk (Einarson, Shuhaiber & Koren 2001; Michalak et al. 2017; Yefet et al. 2018).

Patients who were prescribed aminoglycosides and fluoroquinolones received them for the shortest possible duration. Studies state that aminoglycosides should be avoided because of its potential to cause ototoxicity, nephrotoxicity and neuromuscular blockade. Only a few cases of neonatal ototoxicity have been recorded following short-term administration and the drug may be used in pregnant women with careful monitoring (Chow & Jewesson 1985). It is advised that possible risks should be explained, especially in the first trimester. The use of fluoroquinolones in pregnancy remains controversial. In this study, only 5 patients of a total of 184 women received a fluoroquinolone. A systematic review by Yefet et al. (2018) assessed the safety of quinolones in pregnancy and reported no major congenital malformations. The data suggest that although fluoroquinolones were shown to cause possible harmful effects in some animal models, it is usually with higher concentrations. However, in 2016, the FDA updated its warnings for both oral and injectable fluoroquinolones (Acar et al. 2019). The authors illustrated that when the drug is used systemically, it may be associated with disabling and potentially permanent side effects, which can involve the disruption of tendons, joints, muscles and nerves in the foetus. Besides the effects mentioned, it could even induce type 2 diabetes. This was a result of an increase in the number of reports about fluoroquinolone toxicity and long-term complications. The FDA has since introduced significant restrictions to its use (Acar et al. 2019; Michalak et al. 2017; Yefet et al. 2018).

Theoretically, sulphonamides are contraindicated in the third trimester because of the potential risk of neonatal haemolysis, methemoglobinemia and the fear of kernicterus, although practical evidence of this risk is sparse (Sivojelezova et al. 2003). In this study, seven prescriptions for trimethoprim-sulphonamide combinations were dispensed to women who were in the third trimester of pregnancy for an average duration of 22 days for infections of the blood and circulatory system. The use and long duration of this antibiotic might be because of the antibiotics beneficial uses exceeding its risk.

Chloramphenicol (category C) is one of the few antibiotics that is completely avoided in pregnancy because of the risk of neonatal toxicity, particularly grey baby syndrome (Amstey 2000; Chung, Kwok & Chung 2004). It was observed that three patients received this drug for a duration of 28 days, although it was in the form of an eye ointment. There is a very small probability that large amounts of the drug would be absorbed systemically, which could harm the infant (Chung et al. 2004).

According to several studies, the majority of antibiotics are used to treat genitourinary infections in pregnancy (Amann et al. 2006; Ghouri, Hollywood & Ryan 2019; Lee et al. 2019, 2020). Observantly, a large percentage of women in this study were treated predominantly for systemic infections. Infections of the blood and circulatory system were most common, whilst infections of the genitourinary tract were the third most commonly treated. This could be as a result of women attending a tertiary hospital for the treatment of more severe infections.

## Implications and recommendations

There is currently a paucity of data regarding antibiotic use in pregnancy and further research should be made in this field. It is recommended that healthcare professionals keep abreast with the new safety data that are continuously being published and incorporate that information into prescribing practices to ensure the safe use of antibiotics in this vulnerable population. It is also essential that more studies follow up on the side effects that drugs have on pregnant women.

## Limitations

There are a few limitations to this study. This study was conducted in one site, in one town and only included individuals who visited the hospital between January and July 2019 with a moderate study population. The results, therefore, cannot be extrapolated to either the national public or private sector population or the general South African population. There were also very limited patient demographic data that could be extracted from patients’ records. There was inadequate time to follow up on those patients to identify whether their infants were affected by the antibiotic therapy received. Despite these limitations, this study contributes to research in this population group and builds on the evidence that currently exists.

## Conclusion

This study shows that despite the lack of clinical evidence, many classes of antibiotics are used in pregnancy apart from those with a safety record (category B antibiotics). The long-term reliance on antibiotics that are considered safe to use has led to emerging resistance resulting in the use of broad-spectrum or alternative agents where safety has not yet been established. Patients should be educated and encouraged to be a part of the decision-making process. The role of healthcare professionals in risk assessment and evaluation of available evidence for optimal antibiotic selection, dosing, duration of therapy and monitoring is pertinent to improving maternal health.

## Appendix 1: Data collection template

**Site:** Inkosi Albert Luthuli Hospital

**Date:**

**Time:**

**Time period:** January – July 2019

**Collected by:**

**Inclusion Criteria:**

- Female
- Pregnant
- 18 years of age and over
- Treated with an antibiotic

**Table:**

Patient no:	Patients demographics (Age, race, employment status, occupation, educational level, marital status, no. of children)	Inpatient/outpatient	If inpatient: How many days were spent in hospital	Trimester/Gestational period	Diagnosis/reason for administering the chosen antibiotic	Was sensitivity testing performed: Y/N	Prescriber	Generic name of antibiotic/s prescribed E.g.: Amoxicillin	Brand name of antibiotic/s Prescribed Eg: Moxypen	Class of antibiotic/s prescribed	Dosage/strength, frequency and quantity of the antibiotic/s	Dosage form	Duration of treatment	Was the antimicrobial on the formulary list	Reported side effects	Any other complications E.g. HIV positive, Diabetic, Hypertensive, high risk patient, previous miscarriage/pneumonia	Cost of treatment	Other
																		
																		
																		
																		
																		
																		
																		
																		
																		

## References

[CIT0001] Acar, S., Keskin-Arslan, E., Erol-Coskun, H., Kaya-Temiz, T. & Kaplan, Y.C., 2019, ‘Pregnancy outcomes following quinolone and fluoroquinolone exposure during pregnancy: A systematic review and meta-analysis’, *Reproductive Toxicology* 85, 65–74. 10.1016/j.reprotox.2019.02.002 30738954

[CIT0002] Adam, M.P., Polifka, J.E. & Friedman, J.M., 2011, ‘Evolving knowledge of the teratogenicity of medications in human pregnancy’, *American Journal of Medical Genetics Part C: Seminars in Medical Genetics* 157(3), 175–182. 10.1002/ajmg.c.30313 21766440

[CIT0003] Adhikari, M., 2009, ‘Tuberculosis and tuberculosis/HIV co-infection in pregnancy’, *Seminars in Foetal and Neonatal Medicine* 14(4), 234–240. 10.1016/j.siny.2009.02.001 19303830

[CIT0004] Amann, U., Egen-Lappe, V., Strunz-Lehner, C. & Hasford, J., 2006, ‘Antibiotics in pregnancy: Analysis of potential risks and determinants in a large German statutory sickness fund population’, *Pharmacoepidemiology and Drug Safety* 15(5), 327–337. 10.1002/pds.1225 16557603

[CIT0005] Amstey, M.S., 2000, ‘Chloramphenicol therapy in pregnancy’, *Clinical Infectious Diseases* 30(1), 237. 10.1086/313582 10619781

[CIT0006] Anderson, G.D., 2005, ‘Pregnancy-induced changes in pharmacokinetics’, *Clinical Pharmacokinetics* 44(10), 989–1008. 10.2165/00003088-200544100-00001 16176115

[CIT0007] Anitha, B., Malavika, S., Kumar, B. & Ramesh, Y., 2018, ‘Current trends in drugs avoided in pregnancy’, *Journal of Drug Delivery and Therapeutics* 8(6), 342–350. 10.22270/jddt.v8i6.2035

[CIT0008] Blanca-Lopez, N., Jimenez-Rodriguez, T.W., Somoza, M.L., Gomez, E., Al-Ahmad, M., Perez-Sala, D. et al., 2019, ‘Allergic reactions to penicillins and cephalosporins: Diagnosis, assessment of cross-reactivity and management’, *Expert Review of Clinical Immunology* 15(7), 707–721. 10.1080/1744666X.2019.1619548 31161822

[CIT0009] Bookstaver, P.B., Bland, C.M., Griffin, B., Stover, K.R., Eiland, L.S. & McLaughlin, M., 2015, ‘A review of antibiotic use in pregnancy’, *Pharmacotherapy: The Journal of Human Pharmacology and Drug Therapy* 35(11), 1052–1062. 10.1002/phar.1649 26598097

[CIT0010] Broe, A., Pottegard, A., Lamont, R.F., Jorgensen, J.S. & Damkier, P., 2014, ‘Increasing use of antibiotics in pregnancy during the period 2000–2010: Prevalence, timing, category, and demographics’, *BJOG: An International Journal of Obstetrics and Gynaecology* 121(8), 988–996. 10.1111/1471-0528.12806 24754708

[CIT0011] Calder, C.L., O’Hara, H., Tabatabai, M., Maxwell, C.J., Marryshow, S., Ahonkhai, A.A. et al., 2020, ‘Adherence to combination antiretroviral therapy among pregnant women enrolled in a HIV prevention program in rural North-central Nigeria’, *International Journal of Maternal and Child Health and AIDS* 9(1), 81–92. 10.21106/ijma.327 32123632 PMC7031888

[CIT0012] Cherney, K., 2016, *Understanding infections in pregnancy. Healthline: Infections in pregnancy*, viewed 29 March 2019, from https://www.healthline.com/health/pregnancy/infections.

[CIT0013] Chow, A.W. & Jewesson, P.J., 1985, ‘Pharmacokinetics and safety of antimicrobial agents during pregnancy’, *Reviews of Infectious Diseases* 7(3), 287–313. 10.1093/clinids/7.3.287 3895351

[CIT0014] Chung, C.Y., Kwok, A.K.H. & Chung, K.L., 2004, ‘Use of ophthalmic medications during pregnancy’, *Hong Kong Medical Journal* 10(3), 191–196. 15181224

[CIT0015] Cohn, S.E. & Clark, R.A., 2014, ‘Human immunodeficiency virus infection in women’, in *Mandell, Douglas, and Bennett’s principles and practice of infectious diseases*, pp. 1590–1615, Elsevier Inc, Amsterdam.

[CIT0016] Costantine, M., 2014, ‘Physiologic and pharmacokinetic changes in pregnancy’, *Frontiers in Pharmacology* 5, 65. 10.3389/fphar.2014.00065 24772083 PMC3982119

[CIT0017] Crider, K.S., Cleves, M.A., Reefhuis, J., Berry, R.J., Hobbs, C.A. & Hu, D.J., 2009, ‘Antibacterial medication use during pregnancy and risk of birth defects: National Birth Defects Prevention Study’, *Archives of Paediatrics and Adolescent Medicine* 163(11), 978–985. 10.1001/archpediatrics.2009.188 19884587

[CIT0018] Department of Health: Province of KwaZulu Natal, 2017, *Inkosi Albert Luthuli Central Hospital*, viewed 13 May 2020, from http://www.ialch.co.za/.

[CIT0019] Dinur, A.B., Koren, G., Matok, I., Wiznitzer, A., Uziel, E., Gorodischer, R. et al., 2013, ‘Foetal safety of macrolides’, *Antimicrobial Agents and Chemotherapy* 57(7), 3307–3311. 10.1128/AAC.01691-12 23650169 PMC3697347

[CIT0020] Einarson, A., Shuhaiber, S. & Koren, G., 2001, ‘Effects of antibacterials on the unborn child’, *Paediatric Drugs* 3(11), 803–816. 10.2165/00128072-200103110-00003 11735666

[CIT0021] Erickson, M.A. & Banks, W.A., 2019, ‘Age-associated changes in the immune system and blood–brain barrier functions’, *International Journal of Molecular Sciences* 20(7), 1632. 10.3390/ijms20071632 PMC6479894 30986918

[CIT0022] Fan, H., Gilbert, R., O’Callaghan, F. & Li, L., 2020, ‘Associations between macrolide antibiotics prescribing during pregnancy and adverse child outcomes in the UK: Population based cohort study’, *BMJ* 19, 368. 10.1136/bmj.m331 PMC7190043 32075790

[CIT0023] Feibus, K.B., 2008, ‘FDA’s proposed rule for pregnancy and lactation labelling: Improving maternal child health through well-informed medicine use’, *Journal of Medical Toxicology* 4(4), 284–288. 10.1007/BF03161214 19031382 PMC3550120

[CIT0024] Food and Drug Administration, 2017, *Pregnancy, lactation, and reproductive potential: Labelling for human prescription drug and biological products-content and format: Guidance for industry*, US Food and Drug Administration, United States, December 2014.

[CIT0025] Food and Drug Administration, 2019, *Pregnancy and lactation labelling final rule*, viewed 02 October 2019, from https://www.fda.gov.

[CIT0026] Frederiksen, M.C., 2001, ‘Physiologic changes in pregnancy and their effect on drug disposition’, *Seminars in Perinatology* 25(3), 120–123. 10.1053/sper.2001.24565 11453606

[CIT0027] Ghouri, F., Hollywood, A. & Ryan, K., 2019, ‘Urinary tract infections and antibiotic use in pregnancy-qualitative analysis of online forum content’, *BMC Pregnancy and Childbirth* 19(1), 1–8. 10.1186/s12884-019-2451-z 31409404 PMC6693226

[CIT0028] Heikkilä, A., Renkonen, O.V. & Erkkola, R., 1992, ‘Pharmacokinetics and transplacental passage of imipenem during pregnancy’, *Antimicrobial Agents and Chemotherapy* 36(12), 2652–2655. 10.1128/AAC.36.12.2652 1482132 PMC245522

[CIT0029] Heikkila, A. & Erkkola, R., 1994a, ‘Review of B-lactam antibiotics in pregnancy’, Clinical Pharmacokinetics 27(1), 49–62. 10.2165/00003088-199427010-00005 7955771

[CIT0030] Heikkila, A. & Erkkola, R., 1994b, ‘B-lactam antibiotics in pregnancy-pharmacokinetic aspects’, Journal of Obstetrics and Gynaecology 14(Suppl. 2), 99–102. 10.3109/01443619409015483

[CIT0031] Knothe, H. & Dette, G.A., 1986, ‘Antibiotics in pregnancy: Toxicity and teratogenicity’, *Obstetrical and Gynaecological Survey* 41(1), 31–33. 10.1097/00006254-198601000-00007

[CIT0032] Kuperman, A.A. & Koren, O., 2016, ‘Antibiotic use during pregnancy: How bad is it’, *BMC Medicine* 14(1), 91. 10.1186/s12916-016-0636-0 27312712 PMC4911692

[CIT0033] Lamont, H.F., Blogg, H.J. & Lamont, R.F., 2014, ‘Safety of antimicrobial treatment during pregnancy: A current review of resistance, immunomodulation and teratogenicity’, *Expert Opinion on Drug Safety* 13(12), 1569–1581. 10.1517/14740338.2014.939580 25189188

[CIT0034] Ledger, W.J. & Blaser, M.J., 2013, ‘Are we using too many antibiotics during pregnancy’, *BJOG: An International Journal of Obstetrics and Gynaecology* 120(12), 1450–1452. 10.1111/1471-0528.12371 24118809 PMC4492536

[CIT0035] Lee, A.C., Mullany, L.C., Koffi, A.K., Rafiqullah, I., Khanam, R., Folger, L.V. et al., 2020, ‘Urinary tract infections in pregnancy in a rural population of Bangladesh: Population-based prevalence, risk factors, etiology, and antibiotic resistance’, *BMC Pregnancy and Childbirth* 20(1), 1–11. 10.1186/s12884-019-2665-0 PMC6938613 31892316

[CIT0036] Lee, A.C., Mullany, L.C., Quaiyum, M., Mitra, D.K., Labrique, A., Christian, P. et al., 2019, ‘Effect of population-based antenatal screening and treatment of genitourinary tract infections on birth outcomes in Sylhet, Bangladesh (MIST): A cluster-randomised clinical trial’, *The Lancet Global Health* 7(1), 148–159. 10.1016/S2214-109X(18)30441-8 PMC6293967 30554751

[CIT0037] Loto, O.M. & Awowole, I., 2012, ‘Tuberculosis in pregnancy: A review’, *Journal of Pregnancy* 2012, Article ID 379271. 10.1155/2012/379271 PMC3206367 22132339

[CIT0038] Mendelson, M., Brink, A., Gouws, J., Mbelle, N., Naidoo, V., Pople, T. et al., 2018, ‘The One Health stewardship of colistin as an antibiotic of last resort for human health in South Africa’, *The Lancet Infectious Diseases* 18(9), 288–294. 10.1016/S1473-3099(18)30119-1 29673734

[CIT0039] Mepham, S., Zondi, Z., Mbuyazi, A., Mkhwanazi, N. & Newell, M.L., 2011, ‘Challenges in PMTCT antiretroviral adherence in northern KwaZulu-Natal, South Africa’, *AIDS Care* 23(6), 741–747. 10.1080/09540121.2010.516341 21293987

[CIT0040] Michalak, K., Sobolewska-Wlodarczyk, A., Wlodarczyk, M., Sobolewska, J., Wozniak, P. & Sobolewski, B., 2017, ‘Treatment of the fluoroquinolone-associated disability: The pathobiochemical implications’, *Oxidative Medicine and Cellular Longevity* 2017, Article ID 8023935. 10.1155/2017/8023935 PMC5632915 29147464

[CIT0041] Nahum, G.G., Uhl, K. & Kennedy, D.L., 2006, ‘Antibiotic use in pregnancy and lactation: What is and is not known about teratogenic and toxic risks’, *Obstetrics and Gynaecology* 107(5), 1120–1138. 10.1097/01.AOG.0000216197.26783.b5 16648419

[CIT0042] Ntlantsana, V., Hift, R.J. & Mphatswe, W.P., 2019, ‘HIV viraemia during pregnancy in women receiving preconception antiretroviral therapy in KwaDukuza, KwaZulu-Natal’, *Southern African Journal of HIV Medicine* 20(1), 1–8. 10.4102/sajhivmed.v20i1.847 PMC6494933 31061722

[CIT0043] Ramsey, P.S., Vaules, M.B., Vasdev, G.M., Andrews, W.W. & Ramin, K.D., 2003, ‘Maternal and transplacental pharmacokinetics of azithromycin’, *American Journal of Obstetrics and Gynaecology* 188(3), 714–718. 10.1067/mob.2003.141 12634646

[CIT0044] Rossiter, D. (ed.), 2020, *South African medicines formulary*, 13th edn., Health and Medical Publishing Group, Cape Town.

[CIT0045] Santos, F., Oraichi, D. & Bérard, A., 2010, ‘Prevalence and predictors of anti-infective use during pregnancy’, *Pharmacoepidemiology and Drug Safety* 19(4), 418–427. 10.1002/pds.1915 20119971

[CIT0046] Sarkar, M., Woodland, C., Koren, G. & Einarson, A.R., 2006, ‘Pregnancy outcome following gestational exposure to azithromycin’, *BMC Pregnancy and Childbirth* 6(1), 1–5. 10.1186/1471-2393-6-18 PMC1481555 16734900

[CIT0047] Shaabaan, L.A., Zaeri, A.Y., Othman, E.E., Qumiri, A.A., Towiargi, R.S., Alnosair, B.A. et al., 2020, ‘Antibiotic use in the first trimester of pregnancy’, *EC Microbiology* 1, 1–7.

[CIT0048] Sivojelezova, A., Einarson, A., Shuhaiber, S., Koren, G. & Team, M., 2003, ‘Trimethoprim-sulfonamide combination therapy in early pregnancy’, *Canadian Family Physician* 49(9), 1085–1086. 14526858 PMC2214286

[CIT0049] Stokholm, J., Schjorring, S., Pedersen, L., Bischoff, A.L., Folsgaard, N., Carson, C.G. et al., 2013, ‘Prevalence and predictors of antibiotic administration during pregnancy and birth’, *PLoS One* 8(12), e82932. 10.1371/journal.pone.0082932 24340068 PMC3858309

[CIT0050] Thinkhamrop, J., Hofmeyr, G.J., Adetoro, O., Lumbiganon, P. & Ota, E., 2015, ‘Antibiotic prophylaxis during the second and third trimester to reduce adverse pregnancy outcomes and morbidity’, *Cochrane Database of Systematic Reviews* 1(4). 10.1002/14651858.CD002250.pub2 25621770

[CIT0051] USAID, 2012, *How to investigate antimicrobial use in hospitals: Selected indicators*, pp.8–31, Strengthening Pharmaceutical Systems Program, Management Sciences for Health, Arlington, VA, viewed 20 May 2019, from http://www.msh.org/projects/sps/SPS-Documents/upload/Indicator-based-Study-on-Hospital-Antimicrobial-Use_Manual_Final.pdf.

[CIT0052] Van de Mheen, L., Smits, S.M., Terpstra, W.E., Leyte, A., Bekedam, D.J. & Van den Akker, E.S., 2014, ‘Haemolytic anaemia after nitrofurantoin treatment in a pregnant woman with G6PD deficiency’, *Case Reports* 2014, bcr2013010087. 10.1136/bcr-2013-010087 PMC4025214 24789148

[CIT0053] Vidal, A.C., Murphy, S.K., Murtha, A.P., Schildkraut, J.M., Soubry, A., Huang, Z. et al., 2013, ‘Associations between antibiotic exposure during pregnancy, birth weight, and aberrant methylation at imprinted genes among offspring’, *International Journal of Obesity* 37(7), 907–913. 10.1038/ijo.2013.47 23609933 PMC3705584

[CIT0054] Walsh, C.T. & Wright, G., 2005, ‘Introduction: Antibiotic resistance’, *Chemical Reviews* 105(2), 391–393. 10.1021/cr030100y 15700949

[CIT0055] Yefet, E., Schwartz, N., Chazan, B., Salim, R., Romano, S. & Nachum, Z., 2018, ‘The safety of quinolones and fluoroquinolones in pregnancy: A meta-analysis’, *BJOG: An International Journal of Obstetrics and Gynaecology* 125(9), 1069–1076. 10.1111/1471-0528.15119 29319210

